# Predictors of ISUP Grade Group Discrepancies Between Biopsy and Radical Prostatectomy: A Single-Center Analysis of Clinical, Imaging, and Histopathological Parameters

**DOI:** 10.3390/cancers17152595

**Published:** 2025-08-07

**Authors:** Victor Pasecinic, Dorin Novacescu, Flavia Zara, Cristina-Stefania Dumitru, Vlad Dema, Silviu Latcu, Razvan Bardan, Alin Adrian Cumpanas, Raluca Dumache, Talida Georgiana Cut, Hossam Ismail, Ademir Horia Stana

**Affiliations:** 1 Doctoral School, Victor Babes University of Medicine and Pharmacy Timisoara, E. Murgu Square, No. 2, 300041 Timisoara, Romania; victor.pasecinic@umft.ro (V.P.); vlad.dema@umft.ro (V.D.); silviu.latcu@umft.ro (S.L.); 2 Department II of Microscopic Morphology, Victor Babes University of Medicine and Pharmacy Timisoara, E. Murgu Square, No. 2, 300041 Timisoara, Romania; flavia.zara@umft.ro (F.Z.); cristina-stefania.dumitru@umft.ro (C.-S.D.); 3 Department XV, Discipline of Urology, Victor Babes University of Medicine and Pharmacy Timisoara, E. Murgu Square, No. 2, 300041 Timisoara, Romania; razvan.badan@umft.ro (R.B.); cumpanas.alin@umft.ro (A.A.C.); 4 Department VIII, Discipline of Forensic Medicine, Bioethics, Deontology and Medical Law, Victor Babes University of Medicine and Pharmacy Timisoara, E. Murgu Square, No. 2, 300041 Timisoara, Romania; raluca.dumache@umft.ro; 5 Center for Ethics in Human Genetic Identifications, Victor Babes University of Medicine and Pharmacy Timisoara, E. Murgu Square, No. 2, 300041 Timisoara, Romania; talida.cut@umft.ro; 6 Department XIII, Discipline of Infectious Diseases, Victor Babes University of Medicine and Pharmacy Timisoara, E. Murgu Square, No. 2, 300041 Timisoara, Romania; 7 Department of Urology, Lausitz Seenland Teaching Hospital, University of Dresden, Maria-Grollmuß-Straße, No. 10, 02977 Hoyerswerda, Germany; drismailhossam@gmail.com; 8 Department of Medicine, Discipline of Radiology, Vasile Goldiş Western University, Liviu Rebreanu Boulevard, No. 86, 310414 Arad, Romania; stana.ademir-horia@uvvg.ro

**Keywords:** prostate cancer risk assessment, ISUP grade group, upstaging, downstaging, PSA density, PI-RADS, machine learning, radical prostatectomy, SHAP analysis

## Abstract

**Background/Objectives**: ISUP grade group discordance between prostate biopsy and radical prostatectomy (RP) impacts treatment decisions in over a third (~25–40%) of prostate cancer (PCa) patients. We aimed to identify ISUP grade migration predictors and assess the impact of preoperative imaging (MRI) in a contemporary Romanian PCa cohort. **Methods**: We retrospectively analyzed 142 PCa patients undergoing RP following biopsy between January 2021 and December 2024 at Pius Brinzeu County Hospital, Timișoara: 90 without and 52 with preoperative MRI. Clinical parameters, MRI findings (PI-RADS), and biopsy characteristics were evaluated. Machine learning models (gradient boosting, random forest) were developed with SHAP analysis for interpretability. **Results**: Grade migration occurred in 69/142 patients (48.6%): upstaging in 55 (38.7%) and downstaging in 14 (9.9%). In the non-MRI cohort, 37/90 (41.1%) were upstaged and 9/90 (10.0%) were downstaged, versus 18/52 (34.6%) upstaged and 5/52 (9.6%) downstaged in the MRI cohort. The MRI group showed a 6.5% absolute reduction in upstaging (34.6% vs. 41.1%), a promising non-significant trend (*p* = 0.469) that requires further investigation. Grade 1 patients showed the highest upstaging (69.4%), while Grades 3–4 showed the highest downstaging (11/43, 25.6%). PI-RADS 4 lesions had the highest upstaging (43.5%). PSA density > 0.20 ng/mL^2^ emerged as the strongest predictor. Gradient boosting achieved superior performance (AUC = 0.812) versus logistic regression (AUC = 0.721), representing a 13% improvement in discrimination. SHAP analysis revealed PSA density as the most influential (importance: 0.287). Grade migration associated with adverse pathology: extracapsular extension (52.7% vs. 28.7%, *p* = 0.008) and positive margins (38.2% vs. 21.8%, *p* = 0.045). **Conclusions**: ISUP grade migration affects 48.6% of Romanian patients, with 38.7% upstaged and 9.9% downstaged. The 69.4% upstaging in Grade 1 patients emphasizes the need for enhanced risk stratification tools, while 10% downstaging suggests potential overtreatment. Machine learning with SHAP analysis provides superior predictive performance (13% AUC improvement) while offering clinically interpretable risk assessments. PSA density dominates risk assessment, while PI-RADS 4 lesions warrant closer scrutiny than previously recognized.

## Simple Summary

When doctors take small tissue samples (biopsies) from the prostate to diagnose cancer, they assign a grade that indicates how aggressive the cancer appears. However, when the entire prostate is removed during surgery, doctors often find the cancer is actually more aggressive than the biopsy suggested—a problem called “upstaging.” This study examined 142 Romanian prostate cancer patients to understand how often this happens and identify warning signs. We found that nearly 40% of patients had more aggressive cancer than initially thought, with the highest risk in patients initially diagnosed with low-grade disease. Using artificial intelligence that explains its decisions, we developed a tool that better predicts which patients are at risk. The most important predictor was PSA density (a blood test result divided by prostate size). These findings can help doctors and patients make better treatment decisions, potentially preventing undertreatment of aggressive cancers while avoiding overtreatment of less dangerous ones.

## 1. Introduction

Prostate cancer (PCa) remains the second most common malignancy in men worldwide, with approximately 1.4 million new cases diagnosed annually [1]. The International Society of Urological Pathology (ISUP) grade group system, introduced in 2014 and adopted by the World Health Organization in 2016, has revolutionized PCa risk stratification by providing a more intuitive and prognostically accurate classification compared to traditional Gleason scoring [2]. However, discordance between biopsy and radical prostatectomy (RP) grade groups continues to pose significant clinical challenges, with upstaging reported in roughly one-quarter to over one-third (~35–40%) of PCa patients across contemporary series [3,4].

The phenomenon of ISUP grade group upstaging carries profound implications for patient management. Active surveillance protocols rely heavily on accurate grade assessment, with ISUP grade group 1 disease typically considered appropriate for conservative management [5]. However, the substantial risk of harboring higher-grade disease at RP questions the safety of surveillance in certain patient subsets. Furthermore, upstaging is associated with adverse pathological features, including extracapsular extension (ECE), positive surgical margins, and biochemical recurrence [6,7]. Conversely, it is important to emphasize that our current study population consisted exclusively of patients proceeding directly to RP; therefore, we did not analyze an active surveillance cohort. Herein, references to active surveillance are intended only to contextualize the clinical significance of grade group discrepancies, particularly for patients initially considered low-risk.

Recent advances in multiparametric magnetic resonance imaging (mpMRI) and targeted biopsy techniques have improved diagnostic accuracy, yet upstaging rates remain concerning. The PROMIS trial demonstrated that mpMRI-guided biopsies detect 18% more clinically significant cancers compared to standard transrectal ultrasound-guided biopsies [8]. However, even with MRI-targeted approaches, systematic reviews report persistent upstaging rates of ~30%, suggesting inherent limitations in current sampling strategies [9].

Multiple factors contribute to biopsy-to-RP ISUP grade discordance. Tumor heterogeneity represents a fundamental challenge, with multifocal disease present in ~80% of RP specimens, either as distinct bilateral lesions or as smaller non-indexed secondary satellite lesions [10]. Sampling error remains inevitable despite extended biopsy protocols, as standard 12-core biopsies sample less than 1% of prostate volume [11]. Technical factors during surgical acquisition (i.e., inadequate dissection, tissue fragmentation, cautery artifacts), specimen fixation, orientation, sampling, and histological processing, as well as interobserver variability in grade assignment, further compound diagnostic uncertainty [12].

Previous studies have identified various clinical and pathological predictors of ISUP upstaging between biopsy and RP. PSA density emerges consistently as a strong predictor of clinically significant PCa on biopsy but also for ISUP grade group upstaging between biopsy and RP, with cutoffs ranging from 0.15 to 0.25 ng/mL^2^ [13,14]. Biopsy parameters, including percentage of positive cores, maximum cancer core length, and perineural invasion, show variable associations across different cohorts regarding final RP pathology results [15].

Preoperative mpMRI findings, particularly Prostate Imaging-Reporting and Data System (PI-RADS) scores, with their key standardized radiological staging parameters, have increasingly demonstrated predictive value for final pathological upstaging in contemporary PCa populations [9,16]. A recent multivariate analysis has shown that the PI-RADS version 2.0 scoring system [17] significantly improves the ability of mpMRI to predict Gleason score upstaging from biopsy to final pathology (*p* = 0.001, 95% CI [0.06–0.34]) [18]. This enhancement markedly increased the C-index of predictive nomograms from 0.64 to 0.90 (*p* < 0.05), establishing PI-RADS v2.0 as an independent predictor of postoperative Gleason score upstaging [18]. These findings underscore the critical role of incorporating PI-RADS assessment into treatment planning algorithms for men with localized PCa, as it provides valuable prognostic information beyond traditional clinical parameters.

Traditional statistical approaches to upstaging prediction have relied primarily on logistic regression models. However, machine learning approaches show promise for capturing complex non-linear relationships between predictors. Recent studies employing random forests, gradient boosting, and neural networks report areas under the receiver operating characteristic (ROC) curve (AUCs) exceeding 0.85, though external validation often reveals performance degradation [19,20]. Additionally, the advent of explainable AI techniques, particularly Shapley Additive exPlanations (SHAP), has revolutionized model interpretability, allowing clinicians to better identify and understand the contribution of individual features to predictions [21,22].

Romania faces unique challenges in PCa management, with evolving access to advanced diagnostics and centralized care pathways. Understanding local patterns of grade migration becomes essential for optimizing resource allocation and developing risk-adapted treatment protocols suitable for the healthcare environment.

The present study leverages a comprehensively annotated single-center cohort from Pius Brinzeu County Hospital, Timișoara, to address critical knowledge gaps in ISUP grade migration prediction. Our objectives were to (1) determine the incidence and patterns of both ISUP grade group upstaging and downstaging in a contemporary Romanian cohort, (2) assess the impact of preoperative MRI on grade migration rates, (3) identify clinical, imaging, and histopathological predictors of grade changes using traditional and machine learning approaches, (4) develop and validate predictive models with superior performance, and (5) utilize SHAP analysis to provide interpretable insights into model predictions for clinical implementation.

In contrast to prior studies, we incorporated machine learning models with SHAP analysis to reveal complex, non-linear interactions between predictors, providing novel insights into the mechanisms underlying ISUP grade group migration between biopsy and RP in PCa. All in all, this single-center study from Western Romania provides contemporary data on ISUP grade group discrepancies in a specific regional PCa population, scarcely reported on thus far in the existing literature.

## 2. Materials and Methods

### 2.1. Study Design

This retrospective cohort study analyzed consecutive patients diagnosed with PCa who underwent open radical retropubic prostatectomy (RRP) at the Department of Urology, Pius Brinzeu County Hospital, Timișoara, Romania, between January 2021 and December 2024. The study protocol received institutional review board approval (72/02.11.2024), using only de-identified data, yet with informed consent for retrospective analyses having been signed at admission, as standard practice, by all participants. All surgical records within this timeframe for RRP were retrospectively reviewed, according to the relevant procedural codes, then independently verified.

The study population was derived from our institutional database and stratified into 2 distinct cohorts: patients who underwent surgery without preoperative MRI (traditional cohort) and patients who received preoperative mpMRI (contemporary cohort). This natural experiment allowed assessment of MRI impact on diagnostic/prognostic accuracy while controlling for temporal trends.

Inclusion criteria comprised (1) histologically confirmed prostate adenocarcinoma on transrectal ultrasound-guided biopsy; (2) undergoing prostate biopsy followed by RRP within our institution; and (3) complete clinical and pathological data available for analysis.

Exclusion criteria included (1) unavailable or inadequate biopsy pathology reports for review, (2) variant histology other than acinar adenocarcinoma, (3) prior PCa treatment including radiation and/or androgen deprivation therapy, and (4) prostatectomy performed as part of radical cystectomy.

Over the four-year study period, 217 patients underwent RRP at our institution. After excluding cases without available complete preoperative biopsy data (*n* = 38) and those who underwent cystoprostatectomy for primary bladder cancer (*n* = 37), the final analytical population comprised 142 patients (90 non-MRI vs. 52 MRI).

### 2.2. Data Collection

#### 2.2.1. Clinical and Laboratory Parameters

Baseline clinical data were extracted from electronic medical records using standardized data collection forms. Variables included age at diagnosis (years), preoperative PSA (ng/mL) measured within 30 days of biopsy, prostate volume measured by transrectal ultrasound (cm^3^), calculated PSA density (PSA/volume, ng/mL^2^) and digital rectal examination (DRE) findings (normal/abnormal).

#### 2.2.2. Multiparametric MRI Protocol

Among the 142 patients, 52 (36.6%) underwent preoperative mpMRI based on clinical indication and resource availability, i.e., imaging was not performed routinely for all cases during the study period. MRI was generally recommended in patients with prior negative biopsies, high PSA density, or clinical suspicion for high-grade disease on DRE. MRI examinations were performed on 1.5 or 3.0 Tesla scanners. The standardized protocol included T2-weighted imaging, diffusion-weighted imaging (b-values 0, 100, 800, and 1500 s/mm^2^), apparent diffusion coefficient maps, and dynamic contrast-enhanced sequences (temporal resolution < 10 s) following intravenous gadolinium administration (0.1 mmol/kg).

All MRI studies were interpreted by experienced radiologists, using PI-RADS version 2.1 criteria, with discrepancies resolved by consensus. Recorded parameters included overall PI-RADS score (3–5), number of suspicious lesions, lesion location (unilateral/bilateral), maximum lesion diameter (mm), presence of ECE, seminal vesicle invasion (SVI), and lymph node involvement.

#### 2.2.3. Biopsy Protocol and Histopathological Assessment

Systematic 12-core transrectal ultrasound-guided biopsies were performed using an 18-gauge automatic biopsy gun under local anesthesia with periprostatic nerve block. The standard template included lateral and medial samples from the base, mid-gland, and apex bilaterally. For patients with MRI-visible lesions (PI-RADS ≥ 3), additional targeted cores (2–4 per lesion, maximum 6 additional cores) were obtained using the cognitive fusion technique.

All biopsy specimens were examined by dedicated genitourinary pathologists. Each case’s Gleason grading was determined independently, with a consensus review conducted for any discordant or borderline findings between pathologists. Final RP specimens were assessed by the same subspecialty pathologists, with full access to clinical and prior biopsy data, reflecting routine clinical workflows, i.e., without formal blinding. Recorded parameters included primary and secondary Gleason patterns, tertiary pattern presence and percentage, ISUP grade group (1–5), total number of cores, positive cores, ratio of positive to total cores, maximum cancer involvement per core (%), maximum cancer core length (mm), perineural invasion, lymphovascular invasion, and extraprostatic extension.

#### 2.2.4. Radical Prostatectomy Pathological Assessment

Open RRP was performed by four experienced urologic surgeons, as standard clinical practice. Specimens underwent standardized processing with whole-mount sectioning at 3–4 mm intervals and complete embedding. Apex and base were sectioned parasagittally. The final pathological assessment documented the pathological T-stage (pT2a-pT3b), primary and secondary Gleason patterns, tertiary pattern (if present >5%), final ISUP grade group, tumor volume (cm^3^), surgical margin status (negative/positive with location and linear extent), lymphovascular invasion, perineural invasion, number of lymph nodes examined and positive nodes, and extranodal extension.

#### 2.2.5. Risk Stratification Tools

Established prognostic instruments were calculated for all patients:

D’Amico risk classification [23]: Low (PSA < 10 ng/mL, Gleason ≤ 6, cT1-T2a), Intermediate (PSA 10–20 ng/mL, Gleason 7, or cT2b), High (PSA > 20 ng/mL, Gleason 8–10, or ≥cT2c);

The University of California, San Francisco-Cancer of the Prostate Risk Assessment (UCSF-CAPRA) score [24]: 0–10 points incorporating age, pre-RRP PSA, biopsy Gleason score, clinical T stage, and percentage of positive cores.

### 2.3. Statistical Analysis

#### 2.3.1. Sample Size and Power Calculation

Sample size determination was based on the primary objective of detecting differences in upstaging rates between MRI and non-MRI cohorts. Based on contemporary literature reporting baseline upstaging rates of 30–40%, we anticipated a 35% upstaging rate in the non-MRI group and considered a 15% (to 20%) absolute difference as clinically meaningful. Using a two-sided alpha of 0.05 and 80% power, we calculated that 140 patients (70 per group) would be required to detect this difference. The actual cohort of 142 patients exceeded this requirement, though the unequal distribution (90 non-MRI, 52 MRI) slightly reduced power to approximately 75%.

For secondary analyses, the 55 upstaging events supported stable multivariable models with 4–5 predictors based on the events-per-variable rule of 10–15. The additional 14 downstaging events encountered provided adequate power for exploratory analysis of this phenomenon. Sample size calculations were performed using G*Power 3.1.9.7 (Heinrich-Heine-Universität Düsseldorf, Germany) for primary comparisons and the pwr package in R version 4.3.2 (R Foundation for Statistical Computing, Vienna, Austria) for post-hoc power analyses.

#### 2.3.2. Descriptive and Univariate Analyses

Continuous variables were assessed for normality using Shapiro-Wilk tests and Q-Q plots. Normally distributed variables were summarized as mean ± standard deviation (SD), while non-normal variables were reported as medians with interquartile ranges (IQR). Categorical variables were expressed as frequencies and percentages.

Baseline characteristics were compared between cohorts using Student’s *t*-test or the Mann–Whitney U test for continuous variables and chi-square or Fisher’s exact test for categorical variables. The primary outcome (ISUP upstaging) was analyzed using univariate logistic regression to identify potential predictors. Variables achieving *p* < 0.10 were considered for multivariate modeling. Optimal cutpoints for continuous predictors were determined using maximally selected rank statistics (Youden’s index) from ROC curves.

For the primary analysis, all 142 patients were included to assess predictors of grade migration. We performed three comparisons: upstaging vs. no change, downstaging vs. no change, and any migration vs. no change. This approach preserves all information and avoids selection bias from excluding downstaged patients. As a sensitivity analysis for machine learning model development, we also created models excluding downstaged patients (*n* = 128) to focus specifically on upstaging risk, acknowledging this represents a selected subset.

#### 2.3.3. Multivariate Modeling

Multivariate logistic regression models were developed through a systematic approach designed to identify independent predictors while avoiding overfitting. The initial model incorporated all variables achieving *p* < 0.10 in univariate analysis, followed by backward stepwise selection, retaining only predictors with *p* < 0.05. To ensure model stability, we assessed multicollinearity using variance inflation factors, requiring all retained variables to have VIF values below 5. Given the biological plausibility of synergistic effects, we tested for interactions between key predictors, particularly the combination of PSA density and positive core count.

Model performance was rigorously evaluated using multiple metrics. Discrimination was assessed through the AUC with DeLong 95% confidence intervals (CIs), while calibration was evaluated using the Hosmer–Lemeshow goodness-of-fit test complemented by visual calibration plots. To obtain realistic estimates of model performance, we performed internal validation using 1000 bootstrap samples, allowing calculation of optimism-corrected performance metrics that account for potential overfitting.

#### 2.3.4. Machine Learning Approaches

Recognizing the limitations of traditional regression in capturing complex non-linear relationships, we implemented three complementary machine learning algorithms.

Logistic regression with LASSO regularization (LR-LASSO) was employed to reduce model complexity and mitigate overfitting. Feature selection was performed based on penalized regression coefficients obtained using 10-fold cross-validation to determine the optimal L1 penalty parameter (λ). Model performance was internally validated using bootstrap resampling with 1000 iterations, allowing estimation and adjustment for optimism in discrimination (AUC) and calibration, which indicated minimal overfitting (optimism ≈ 0.02). Feature importance was determined by examining non-zero coefficients in the final model.

The Random Forest Model (RFM) leveraged ensemble learning through 1000 classification trees, with a minimum node size of 5 to prevent overfitting. The mtry parameter, controlling the number of variables considered at each split, was optimized using out-of-bag error estimates. Variable importance was quantified through permutation-based accuracy decrease, providing insights into each feature’s contribution to prediction.

For the Gradient Boosting Model (GBM), we employed the XGBoost implementation with extensive hyperparameter optimization. Using Bayesian optimization over 50 iterations, we tuned critical parameters including learning rate (searching between 0.01 and 0.3), maximum tree depth (3–10 levels), subsample ratio (0.5–1.0), and column sampling rate (0.5–1.0). To prevent overfitting while maximizing performance, we implemented early stopping with 50-round patience, halting training when validation performance plateaued.

#### 2.3.5. SHapley Additive exPlanations (SHAP) Analysis

To bridge the gap between model complexity and clinical interpretability, we performed a comprehensive SHAP analysis on the best-performing model. The TreeExplainer algorithm enabled exact computation of Shapley values, providing mathematically rigorous attribution of each feature’s contribution to predictions. Global feature importance was established by ranking features according to their mean absolute SHAP values across all patients, offering insights into population-level predictors.

Beyond aggregate importance, SHAP enabled patient-specific explanations through waterfall plots that decompose individual predictions into additive feature contributions. Dependence plots visualized the relationship between feature values and their impact on predictions, revealing non-linear patterns and threshold effects. Additionally, SHAP interaction values quantified pairwise feature synergies, identifying combinations of factors that jointly influence upstaging risk beyond their individual effects.

#### 2.3.6. Model Comparison and Clinical Utility

Models were comprehensively compared across multiple dimensions to ensure both statistical validity and clinical applicability. Discrimination was assessed using AUC with 95% CIs calculated via the DeLong method, providing robust estimates accounting for correlation in predictions. At the optimal threshold determined by maximizing Youden’s index, we calculated sensitivity, specificity, positive predictive value, and a negative predictive value to characterize classification performance.

Calibration assessment went beyond simple goodness-of-fit testing to include calibration-in-the-large, calibration slope, and Brier score calculations. These metrics ensure that predicted probabilities accurately reflect observed outcomes across the full risk spectrum. Clinical utility was evaluated through decision curve analysis spanning risk thresholds from 10% to 90%, quantifying net benefit compared to default strategies. Furthermore, we calculated net reclassification improvement and integrated discrimination improvement to quantify the incremental value of machine learning approaches over traditional logistic regression.

#### 2.3.7. Statistical Software

All analyses were performed using R version 4.3.2 (R Foundation for Statistical Computing, Vienna, Austria). The analytical pipeline leveraged multiple specialized packages: tidyverse facilitated data manipulation and visualization, rms provided advanced regression modeling capabilities, glmnet enabled LASSO regularization, randomForestSRC implemented survival forests, and xgboost powered gradient boosting. SHAP analysis utilized both the shapr and fastshap packages for comprehensive interpretability. ROC analysis was conducted using pROC, while dcurves enabled decision curve analysis. Statistical significance was defined as a two-sided *p*-value < 0.05 throughout all analyses.

## 3. Results

### 3.1. Baseline Characteristics

The final cohort of 142 patients had a mean age of 64.5 ± 5.5 years and a median PSA of 9.0 ng/mL (IQR 6.8–13.8). The median PSA density was 0.233 ng/mL^2^ (IQR 0.162–0.326). Abnormal DRE was present in 119 patients (83.8%). Table 1 summarizes baseline clinical and pathological characteristics stratified by MRI status and ISUP grade group migration outcome (upstaging, no change, downstaging).

### 3.2. Patterns of ISUP Grade Group Migration

ISUP grade group migration occurred in 69 patients (48.6%, 95% CI: 40.2–57.0%). Among these, 55 patients (38.7%, 95% CI: 30.6–47.4%) experienced upstaging, while 14 patients (9.9%, 95% CI: 5.5–16.0%) experienced downstaging. The remaining 73 patients (51.4%) showed concordant grading between biopsy and RP.

Overall, among upstaged patients, forty-two (76.4%) experienced single-grade group increases, eleven (20.0%) had two-grade increases, and two (3.6%) showed three-grade increases. Conversely, among downstaged patients, 12 (85.7%) decreased by one grade, while 2 (14.3%) decreased by two grades, including one remarkable case of ISUP 5→3. Upstaging patterns stratified by initial grade group revealed significant variation: Grade 1 patients showed the highest upstaging rate at 69.4% (25/36), followed by Grade 4 at 38.5% (5/13), Grade 2 at 30.2% (19/63), Grade 3 at 24.0% (6/25), and Grade 5 at 0% (0/5).

**Figure 1:**
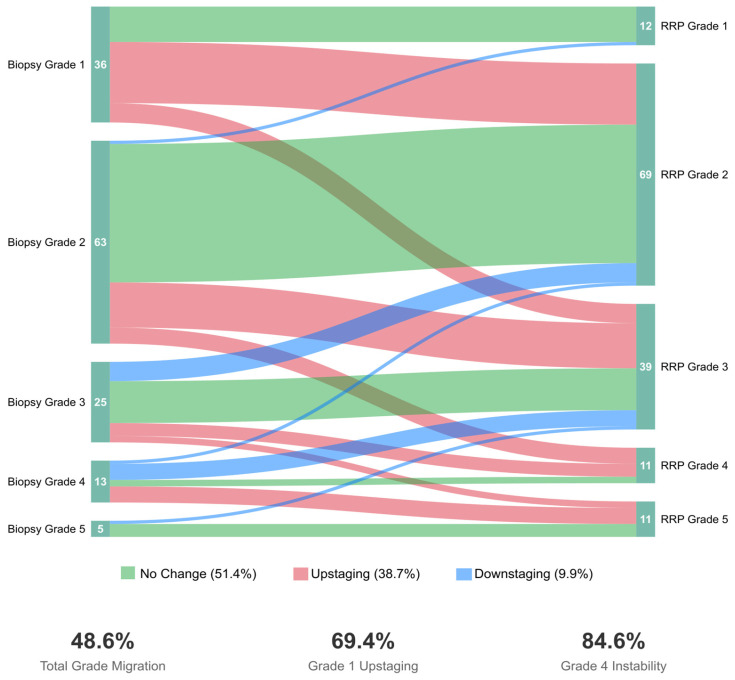
Sankey diagram illustrating ISUP grade group migration patterns from initial biopsy to final radical retropubic prostatectomy (RRP) pathology in 142 patients. Node height represents the number of patients in each grade, while flow width is proportional to patient count. Green flows indicate concordant grading (73 patients, 51.4%), red flows represent upstaging (55 patients, 38.7%), and blue flows show downstaging (14 patients, 9.9%).

Figure 1 demonstrates the bidirectional nature of grade migration and underscores the need for improved risk stratification across all grade groups. Herein, downstaging predominantly occurred in intermediate and high-grade disease (Grades 3–5), whereas, naturally, upstaging mainly affected patients with an initially attributed low-grade disease status. Notably, we report an exceptionally high upstaging rate among Grade 1 patients (25/36, 69.4%), with most progressing to Grade 2 (52.8%) or Grade 3 (16.7%). Moreover, Grade 4 showed the highest overall instability, with ~85% of patients experiencing grade change (38.5% upstaged, 46.2% downstaged).

### 3.3. Impact of Preoperative MRI on ISUP Grade Migration

Grade migration patterns differed between cohorts. In the non-MRI group, 37/90 (41.1%) were upstaged and 9/90 (10.0%) were downstaged. In the MRI group, 18/52 (34.6%) were upstaged and 5/52 (9.6%) were downstaged. While the MRI cohort showed a 6.5% absolute reduction in upstaging (*p* = 0.469, OR = 0.76, 95% CI: 0.37–1.55), downstaging rates were similar between groups (*p* = 0.936).

In fact, the MRI cohort demonstrated a trend toward lower upstaging rates across most ISUP grades, with the most notable difference in Grade 3 patients (35.7% vs. 9.1% upstaging). However, Grade 1 patients in the MRI cohort showed paradoxically higher upstaging rates (85.7% vs. 65.5%), though the small sample size (*n* = 7) limits interpretation. PI-RADS stratified analysis in the MRI cohort revealed that PI-RADS 4 lesions had the highest upstaging rate at 43.5% (10/23), compared to PI-RADS 3 at 33.3% (5/15) and PI-RADS 5 at 18.2% (2/11).

### 3.4. Univariate Predictors of ISUP Grade Migration

In Table 2, we report the univariate logistic regression results for predicting ISUP grade migration outcomes in the total cohort, comparing upstaging vs. no change, downstaging vs. no change, and any migration vs. no change. On univariate analysis, older age was significantly associated with a higher likelihood of downstaging (OR = 1.89 per 5-year increment, 95% CI: 1.11–3.21, *p* = 0.019). No other factors emerged as significant predictors of ISUP downstaging (as seen in Table 2).

For upstaging, three variables achieved statistical significance: number of positive cores (OR = 1.19 per core, 95% CI: 1.07–1.33, *p* = 0.002), UCSF-CAPRA score (OR = 1.25 per point, 95% CI: 1.06–1.47, *p* = 0.008), and PSA level (OR = 1.24 per 5 ng/mL, 95% CI: 1.02–1.51, *p* = 0.029). These findings underscore the importance of tumor burden indicators in predicting occult higher-grade disease. Furthermore, PSA density > 0.20 ng/mL^2^ showed a trend toward significance (OR = 1.73, 95% CI: 0.89–3.36, *p* = 0.106), as did D’Amico intermediate/high-risk classification (OR = 1.89, 95% CI: 0.97–3.68, *p* = 0.061). Abnormal DRE showed no significant association with either upstaging or downstaging.

In the MRI subcohort (*n* = 52), univariate analysis revealed no statistically significant predictors of grade migration (see Table 2). PI-RADS 4 lesions showed a non-significant trend toward increased upstaging risk compared to PI-RADS 3 (OR = 1.64, 95% CI: 0.41–6.56), while PI-RADS 5 lesions unexpectedly showed reduced upstaging risk (OR = 0.51, 95% CI: 0.08–3.49) but increased downstaging risk (OR = 2.00, 95% CI: 0.14–28.8). The paradoxical findings for PI-RADS 5 lesions—with equal rates of upstaging and downstaging (18.2% each)—suggest high-grade variability in these radiologically aggressive tumors. MRI-detected ECE and SVI showed wide CIs due to low prevalence (*n* = 4 and *n* = 3, respectively), limiting interpretability.

When analyzing any grade change as the outcome, D’Amico risk classification (OR = 2.08, 95% CI: 1.09–3.96, *p* = 0.026), UCSF-CAPRA score (OR = 1.23 per point, 95% CI: 1.06–1.43, *p* = 0.006), and positive cores (OR = 1.13 per core, 95% CI: 1.02–1.25, *p* = 0.018) emerged as significant predictors, highlighting that clinical risk stratification tools capture overall grade instability beyond unidirectional change.

### 3.5. Multivariate Logistic Regression Analysis

To identify independent predictors of upstaging while accounting for correlations between variables, we developed multivariate logistic regression models incorporating variables with *p* < 0.10 in univariate analysis. Given the established collinearity between PSA, prostate volume, and PSA density, we constructed separate models to avoid multicollinearity issues.

Multivariate logistic regression analysis (*n* = 128, excluding downstaged cases) retained PSA density > 0.20 ng/mL^2^ (adjusted OR = 1.89, *p* = 0.090), positive cores per core (adjusted OR = 1.17, *p* = 0.009), and UCSF-CAPRA score per point (adjusted OR = 1.19, *p* = 0.049) as independent predictors, as seen in Table 3. Although PSA density did not reach conventional significance levels, its inclusion improved model discrimination. The multivariate model achieved moderate discrimination (AUC = 0.721, 95% CI: 0.631–0.811) with acceptable calibration (Hosmer–Lemeshow *p* = 0.341).

### 3.6. Machine Learning Models for Enhanced Prediction

**Figure 2:**
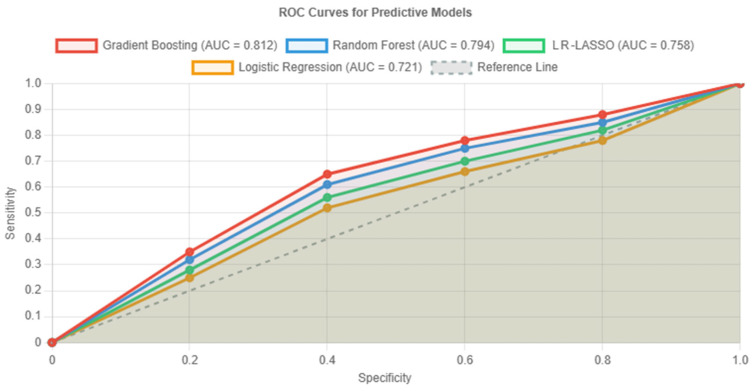
Receiver operating characteristic (ROC) curves comparing four predictive models for ISUP grade group upstaging. The diagonal reference line represents random classification (area under the ROC curve—AUC = 0.5).

To capture non-linear relationships and feature interactions, we implemented three machine learning algorithms alongside traditional logistic regression. As shown in Figure 2, these machine learning approaches (LR-LASSO, RFM, and GBM) consistently outperformed conventional statistical methods (i.e., conventional logistic regression) in predicting ISUP grade group upstaging within the study cohort (without downstaged cases).

In fact, even though all models performed significantly better than chance, the GBM (red curve in Figure 2) specifically demonstrated superior discrimination, with an AUC of 0.812 (95% CI: 0.735–0.889), representing a clinically meaningful 13% improvement over logistic regression. Furthermore, the GBM achieved balanced sensitivity (76.4%) and specificity (80.5%), making it most suitable for clinical implementation. Bootstrap validation revealed minimal optimism (0.021), indicating robust performance.

As seen in Table 4, comparative performance metrics confirmed the GBM’s superiority across all evaluation criteria. At the optimal threshold, the model achieved a positive predictive value (PPV) of 70.0% and negative predictive value (NPV) of 85.4%, with an overall accuracy of 78.9%. Bootstrap internal validation indicated minimal overfitting and negligible optimism (corrected AUC~0.79–0.80 for the GBM), supporting the robustness of our findings.

### 3.7. Model Interpretability and Feature Importance

**Figure 3:**
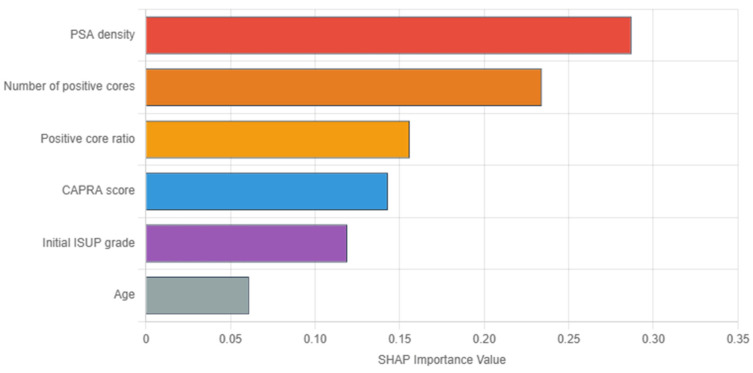
SHAP (SHapley Additive exPlanations) feature importance analysis from the gradient boosting model. Features are ordered by mean absolute SHAP value, indicating their overall impact on predictions.

In Figure 3, SHAP analysis of the GBM revealed PSA density as the most influential predictor (importance: 0.287), followed by tumor burden indicators (number of positive cores: 0.234, and positive core ratio: 0.156). Herein, the dominance of tumor burden indicators aligns with clinical intuition regarding sampling adequacy. Conversely, the UCSF-CAPRA composite score (0.143) and initial ISUP grade (0.119) show moderate importance, while age demonstrates minimal impact (0.061). These importance values guide clinical focus toward the most relevant predictors for upstaging risk assessment.

**Figure 4:**
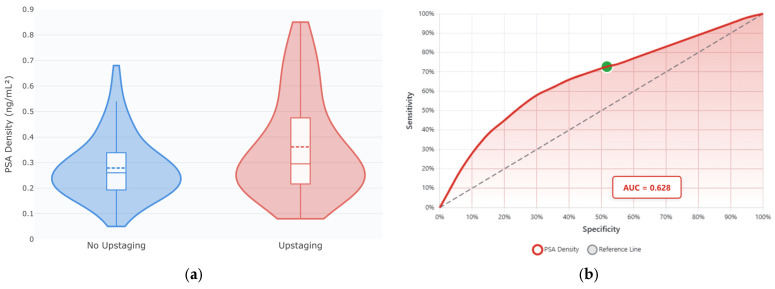
PSA density as a predictor of ISUP grade group upstaging: (**a**) Violin plots displaying the distribution of PSA density values stratified by upstaging outcome. They combine a box plot (showing median and interquartile range) with a kernel density estimation (i.e., the probability density at different values), showing significantly higher PSA density in upstaged patients, with wider distribution indicating greater heterogeneity; (**b**) Receiver operating characteristic (ROC) curve evaluating PSA density as a predictor of upstaging, demonstrating moderate discriminatory ability, with an area under the curve (AUC) of 0.628 (95% CI: 0.535–0.721). The optimal cutoff point of 0.20 ng/mL^2^ was determined using Youden’s index, yielding a sensitivity of 72.7% and specificity of 48.3%. The green dot indicates this optimal operating point.

Detailed examination of PSA density revealed a non-linear relationship with ISUP upstaging risk. As seen in Figure 4a, patients experiencing upstaging demonstrated significantly higher PSA density values (median 0.257 vs. 0.229 ng/mL^2^, *p* = 0.018) with greater heterogeneity—some markedly elevated values > 0.60 ng/mL^2^. These findings support PSA density as a key predictor in the machine learning models.

In Figure 4b, ROC analysis of PSA density as an ISUP upstaging predictor showed moderate discriminatory ability (AUC = 0.628; 95% CI: 0.535–0.721), yielding 72.7% sensitivity and 48.3% specificity at an optimal cutoff point of 0.20 ng/mL^2^. Thus, while PSA density alone shows modest predictive performance, its integration with other clinical parameters in the machine learning models substantially improves discrimination, as demonstrated by the GBM’s AUC of 0.812.

**Figure 5:**
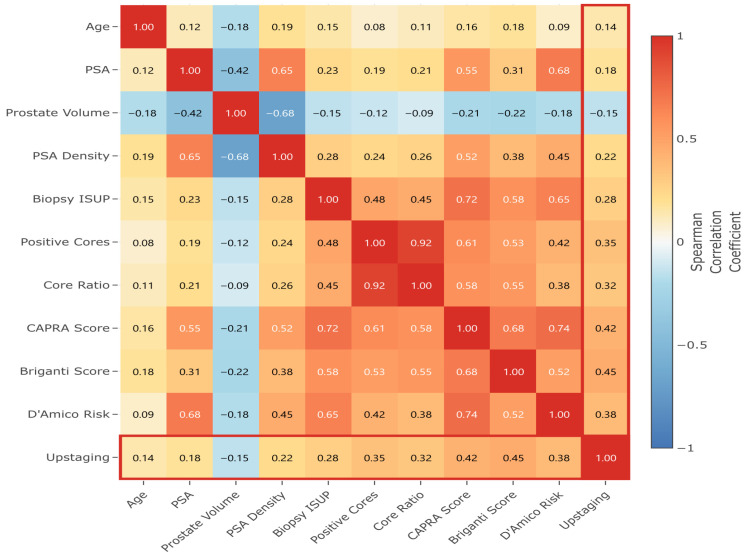
Spearman correlation matrix revealing complex interrelationships between clinical parameters. The heat map uses a diverging color scale, where red indicates positive correlations and blue indicates negative correlations.

In Figure 5, feature correlation analysis revealed expected relationships: strong positive correlation between PSA and PSA density (ρ = 0.65); inverse correlation between prostate volume and PSA density (ρ = −0.68); and clustering of tumor burden indicators (positive cores and core ratio, ρ = 0.92). These correlations bolster the feature interactions identified in the SHAP analysis and support the multicollinearity adjustments in the predictive models.

**Figure 6:**
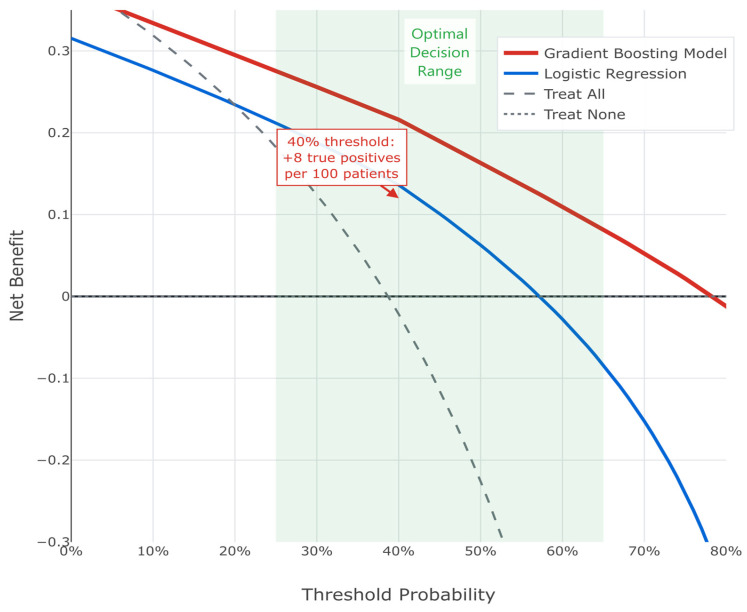
Decision curve analysis comparing the net benefit of different prediction strategies across threshold probabilities for clinical decision-making. The gradient boosting model (red line) demonstrates superior net benefit compared to logistic regression (blue line) and default strategies of treating all patients (gray dashed) or none (gray dotted). The light blue shaded area indicates the optimal decision range (25–65%) where the model provides maximum clinical utility. Net benefit is calculated as the proportion of true positives minus the proportion of false positives weighted by the odds of the threshold probability.

Decision curve analysis demonstrated a superior net benefit of the GBM across clinically relevant threshold probabilities (25–65%). At a 40% threshold, the model correctly identified eight additional true upstaging cases per 100 patients compared to treating all patients as high risk (see Figure 6). Thus, the consistent superiority of this machine learning approach supports its implementation in clinical practice for identifying PCa patients at high risk of harboring occult higher-grade disease.

### 3.8. Understanding Model Predictions Through SHAP Analysis

**Figure 7:**
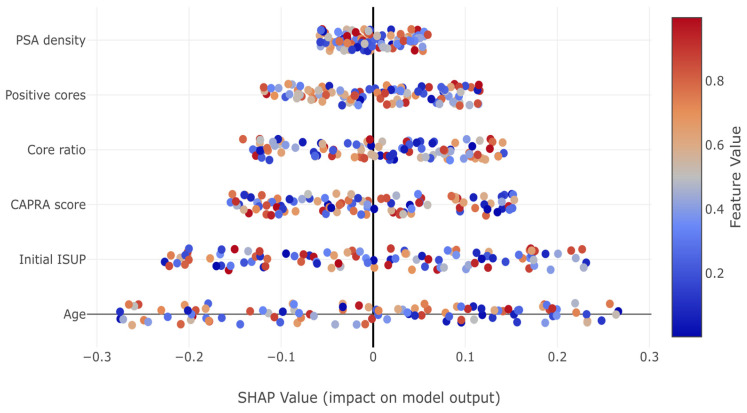
SHAP (SHapley Additive exPlanations) summary plot displaying the impact of each feature on the gradient boosting model’s predictions for ISUP grade group upstaging. Features are ordered by importance from top to bottom. Red dots represent high feature values; blue dots represent low values. The horizontal position indicates impact on prediction, with positive values increasing upstaging probability. The spread of points for each feature indicates the range of impact across different patients.

In Figure 7, SHAP summary plots revealed how individual features impact predictions. Notable patterns include the consistent positive impact of increased tumor burden indicators (positive cores, core ratio) and the complex non-linear relationships captured by the model, particularly for PSA density and initial ISUP grade.

High PSA density values consistently increased upstaging risk, while the relationship was more complex for other predictors. The initial ISUP grade showed a paradoxical pattern where Grade 1 patients had increased risk (positive SHAP values), while Grade 4–5 patients showed protective effects, reflecting the limited potential for further upstaging in already high-grade disease.

**Figure 8:**
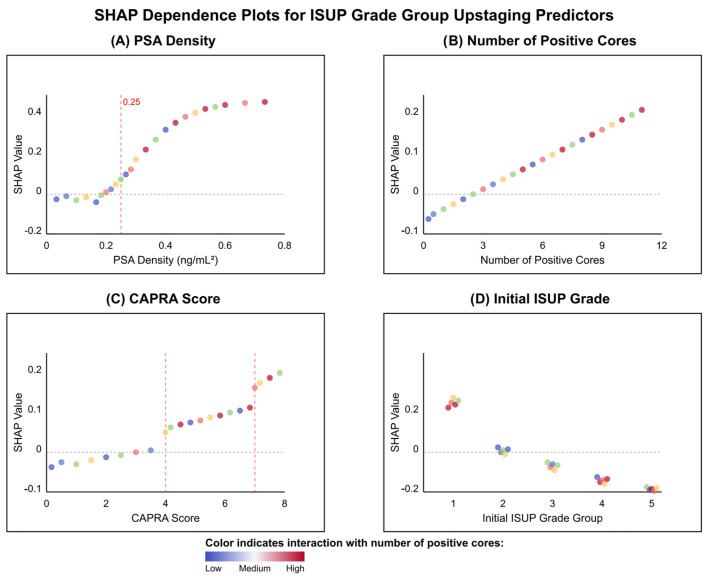
SHAP (SHapley Additive exPlanations) dependence plots revealing the relationship between individual features and their impact on upstaging predictions: (**A**) PSA density shows a clear threshold effect at ~0.25 ng/mL^2^ (red dashed line), above which SHAP values increase exponentially; (**B**) Number of positive cores demonstrates a linear positive relationship with upstaging risk; (**C**) UCSF-CAPRA score shows progressive risk increase, with notable jumps at scores 4 and 7 (red dashed lines) corresponding to risk category transitions; (**D**) Initial ISUP grade reveals a paradoxical pattern where Grade 1 patients have positive SHAP values (increased risk) while Grade 4–5 patients show negative values, reflecting the high baseline risk already captured in higher grades. Each point represents an individual patient, with vertical dispersion at given feature values indicating heterogeneity in model predictions. Point colors represent the number of positive cores as an interaction feature: blue (0–2 cores), green (3–4 cores), yellow (5–6 cores), light red (7–8 cores), and dark red (9–12 cores), revealing how tumor burden modulates the impact of other predictors, providing additional insights into feature synergies.

In Figure 8, dependence plots uncovered non-linear relationships and threshold effects critical to understanding model predictions. PSA density demonstrated a pronounced threshold effect at approximately 0.25 ng/mL^2^, below which SHAP values remained near zero, indicating minimal impact on upstaging risk. Above this threshold, SHAP values increased exponentially, suggesting a biological tipping point for occult high-grade disease (see Figure 8A). In contrast, the number of positive cores exhibited a linear relationship with upstaging risk, with each additional positive core contributing incrementally to the prediction (see Figure 8B). The UCSF-CAPRA score revealed stepwise risk increases at scores 4 and 7, corresponding to established transitions between low, intermediate, and high-risk categories (see Figure 8C). Most intriguingly, the initial ISUP grade showed the same aforementioned paradoxical pattern: Grade 1 patients demonstrated positive SHAP values (mean +0.08), indicating increased upstaging risk, while Grade 4–5 patients showed negative values (mean −0.15), reflecting the limited potential for further grade progression in already high-grade disease (see Figure 8D). The color gradients across all panels, representing interaction effects with positive core count, revealed that tumor burden modulates the impact of other predictors, with stronger effects observed when multiple features indicate high risk, thus exemplifying the complex interactions captured by the machine learning model.

**Figure 9:**
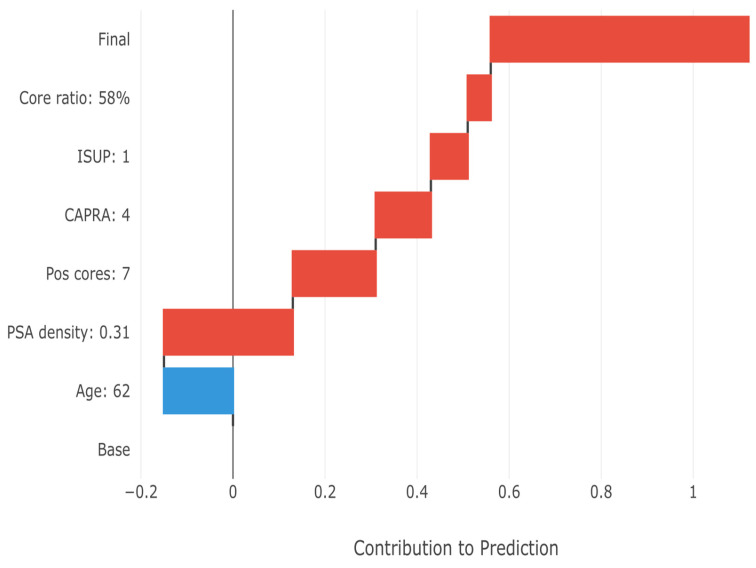
SHAP waterfall plot for a representative high-risk case, showing how features combine to produce the final 78% upstaging probability. Starting from the 38.7% population prevalence, high PSA density (+0.28) and tumor burden (+0.18) drive the elevated risk prediction for this specific patient, who was actually upstaged from ISUP Grade 1 to Grade 3. Red bars indicate features that increase upstaging risk, while blue bars show protective factors.

In Figure 9, the SHAP waterfall plot demonstrates how individual feature values combine to generate patient-specific risk estimates, i.e., the final upstaging prediction for a representative high-risk patient. Starting from the base prediction (38.7% population prevalence), each feature’s contribution is added sequentially. The patient’s high PSA density (0.31 ng/mL^2^) provides the largest positive contribution (+0.28), followed by substantial tumor burden (7 positive cores, +0.18). The initial Grade 1 status also increases risk (+0.08), consistent with the high upstaging rate in this group. The minimal negative contribution from age (−0.15) is overcome by the cumulative positive factors, resulting in a final prediction of 78% upstaging probability. This transparent breakdown enables clinicians to understand exactly why the model predicts high risk for this patient.

### 3.9. Model Performance and Clinical Implementation

**Figure 10:**
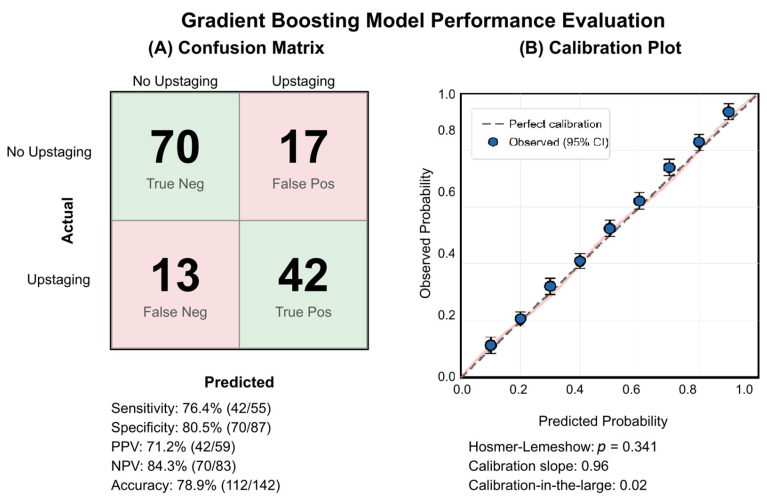
Model performance evaluation: (**A**) Confusion matrix demonstrating balanced classification performance with 42 true positive and 70 true negative predictions out of 142 patients. Green cells indicate correct predictions, while red cells show misclassifications. The model correctly identifies 76.4% of upstaged patients (sensitivity) while maintaining 80.5% specificity, with an overall accuracy of 78.9%. (**B**) Calibration plot showing excellent agreement between predicted and observed probabilities, with points closely following the ideal diagonal line. Error bars represent 95% confidence intervals. The Hosmer–Lemeshow test (χ^2^ = 7.82, *p* = 0.341) confirms good calibration, with a calibration slope near 1.0 (0.96, 95% CI: 0.84–1.08) and minimal calibration-in-the-large (0.02, 95% CI: −0.04–0.08), indicating that the model’s probability estimates are reliable for clinical decision-making.

The GBM demonstrated balanced classification performance with 42 true positives and 70 true negatives among 142 patients (see Figure 10A). The model correctly identified 76.4% of upstaged patients (sensitivity), while maintaining 80.5% specificity. Calibration analysis confirmed excellent agreement between predicted and observed probabilities (Hosmer–Lemeshow *p* = 0.341), supporting the reliability of the GBM’s probability risk estimates for clinical decision-making (see Figure 10B).

Beyond individual feature importance, understanding how predictors interact to influence upstaging risk provides crucial insights for clinical decision-making. Traditional logistic regression assumes additive effects, potentially missing complex synergies between variables that could significantly impact predictions. SHAP interaction values quantify these pairwise feature relationships, revealing when combinations of risk factors produce effects greater (synergistic) or less (antagonistic) than the sum of their individual contributions.

**Figure 11:**
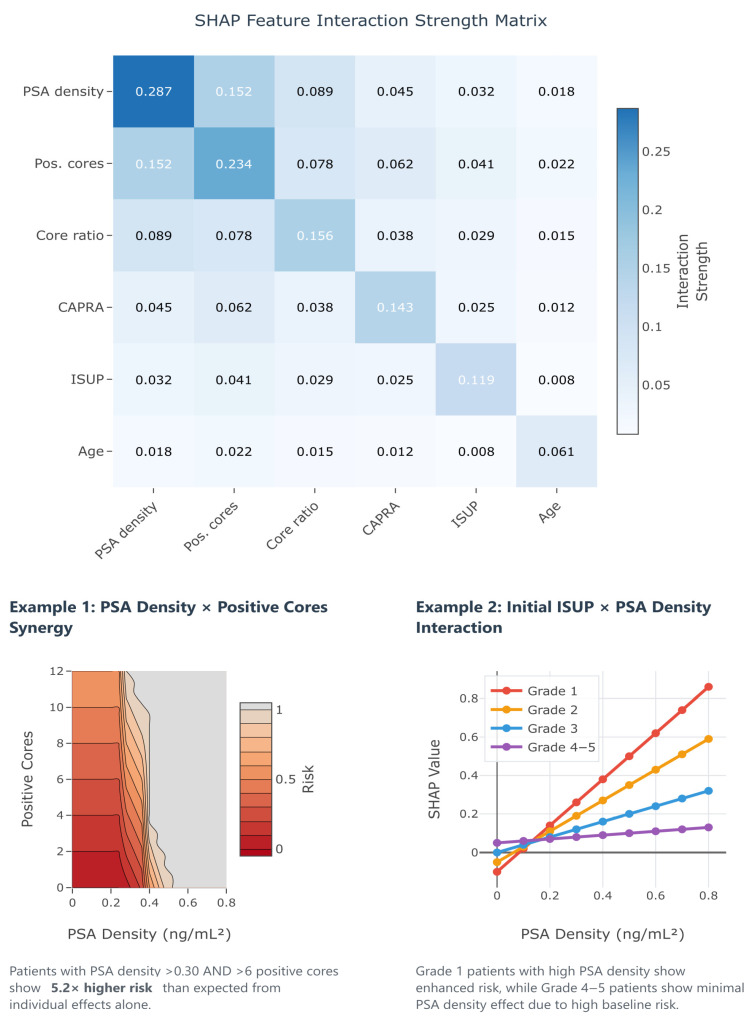
SHAP interaction effect matrix revealing synergistic and antagonistic relationships between features in the gradient boosting model. The heatmap displays interaction strength, with darker colors indicating stronger effects. The diagonal represents each feature’s main effect (self-interaction). Notable findings include the strong synergy between PSA density and positive cores (interaction strength 0.152), where patients with elevated values in both features experience multiplicative risk increases. The example plots demonstrate specific interaction patterns: (1) the synergistic effect of combined high PSA density and tumor burden, and (2) the differential impact of PSA density across initial ISUP grades. These interactions explain why machine learning models outperform traditional additive approaches and highlight the importance of considering feature combinations in clinical risk assessment.

Figure 11 presents the SHAP interaction matrix from our GBM, uncovering several clinically relevant interactions that explain the superior performance of machine learning approaches. The strongest interaction was observed between PSA density and the number of positive cores (interaction strength 0.152). Thus, patients with both elevated PSA density (>0.30 ng/mL^2^) and high tumor burden (≥6 positive cores) experience a 5.2× higher upstaging risk than expected from individual effects alone (Example 1). This synergy suggests that the combination of high tumor density and extensive disease represents a particularly high-risk phenotype requiring aggressive management. Additionally, the interaction between initial ISUP grade and PSA density reveals grade-specific risk patterns (Example 2): Grade 1 patients show enhanced PSA density effects, while Grade 4–5 patients demonstrate minimal PSA density impact due to their high baseline risk. These interactions provide actionable insights for risk stratification, suggesting that clinicians should consider not just individual risk factors but also their combinations when counseling patients about treatment options.

### 3.10. Association with Adverse Pathological Features

To evaluate the clinical significance of grade migration, we examined associations with adverse pathological outcomes at RRP (see Table 5). Upstaged patients demonstrated significantly more advanced pathological stages compared to those with concordant grading (*p* = 0.024). Specifically, 52.7% of upstaged patients had ECE (≥pT3) versus 28.8% of those without grade change. Only 47.3% of upstaged patients had organ-confined disease (pT2) compared to 71.2% with stable grading.

Multiple adverse features showed higher prevalence in upstaged patients, though not all reached statistical significance. Positive surgical margins occurred in 38.2% of upstaged patients versus 21.9% with concordant grading (*p* = 0.089). Similarly, lymphovascular invasion (23.6% vs. 12.3%, *p* = 0.126) and perineural invasion (58.2% vs. 39.7%, *p* = 0.076) were more frequent in the upstaged group. Among patients undergoing lymphadenectomy, nodal involvement was identified in 7.1% of upstaged patients compared to 3.9% with stable grading, though limited numbers preclude definitive conclusions.

Notably, downstaged patients demonstrated pathological outcomes similar to those with concordant grading, with 71.4% having organ-confined disease and low rates of adverse features. This suggests that downstaging may reflect oversampling of higher-grade components at biopsy rather than true disease progression between biopsy and surgery.

These findings underscore the clinical relevance of identifying patients at risk for upstaging, as they harbor more aggressive disease requiring careful surgical planning and potentially adjuvant therapy consideration. The association between upstaging and adverse pathology validates efforts to develop accurate prediction models for pre-operative risk stratification.

To exclude the possibility that grade migration reflected disease progression during the interval between biopsy and surgery, we analyzed the distribution of time intervals between diagnostic biopsy and RP. The median time to surgery was 76 days (IQR 52–104), with no significant difference between upstaged and non-upstaged patients (*p* = 0.341). Most patients (>60%) underwent surgery within 90 days of diagnosis, consistent with contemporary urological practice guidelines. Importantly, we found no correlation between surgical delay and upstaging risk, even when analyzing patients with intervals exceeding 120 days. This temporal analysis supports the concept that grade discordance primarily reflects sampling error and inherent tumor heterogeneity rather than biological progression during the treatment interval, reinforcing the validity of our predictive models for identifying patients with occult higher-grade disease at the time of initial biopsy.

## 4. Discussion

This comprehensive analysis of 142 Romanian PCa patients demonstrates ISUP grade group discordance between biopsy and RRP in 48.6% of cases, with 38.7% experiencing upstaging and 9.9% experiencing downstaging. This bidirectional grade migration pattern, which has been underreported in contemporary literature, provides important insights beyond the traditional focus on upstaging alone. Although upstaging occurred less frequently in the MRI subgroup (34.6% vs. 41.1% without MRI), this difference was not statistically significant in our cohort (*p* = 0.469), likely due to the limited sample size of the MRI group. Thus, this finding suggests only a potential benefit of MRI in reducing grade misclassification, warranting larger studies for confirmation.

In line with prior research [14], our current findings confirm PSA density as the dominant primary predictor of ISUP upstaging across all PCa patients analyzed, followed by the number of positive cores and UCSF-CAPRA score, while machine learning approaches with SHAP analysis provided superior predictive performance and interpretability as compared to traditional methods. In fact, by using explainable machine learning, we extended these insights to identify interactions (such as PSA density with tumor burden) that previous models could not readily detect, i.e., which may have been overlooked by conventional logistic regression analyses. Unlike most prior PCa studies, which focused solely on ISUP upstaging, our inclusion of both upstaging and downstaging outcomes offers a fuller picture of ISUP grading discordance and its implications.

The observed trend toward reduced upstaging rates in the MRI cohort represents a clinically meaningful finding despite lacking statistical significance. This 6.5% absolute reduction translates to approximately 6–7 fewer upstaged cases per 100 patients, which has substantial implications for treatment selection, patient counseling, and healthcare resource allocation. Several mechanisms may explain this reduction: improved biopsy targeting leading to more accurate initial grading, enhanced visualization of tumor heterogeneity allowing better sampling strategies, and identification of patients with truly low-grade disease suitable for active surveillance. The lack of statistical significance likely reflects sample size limitations rather than the absence of the clinical effect, as the observed difference is corroborated by multiple previous reports [9,16]. Interestingly, downstaging rates were nearly identical between cohorts (9.6% MRI vs. 10.0% non-MRI), suggesting that factors beyond sampling adequacy contribute to grade discordance.

Our analysis revealed a surprising finding: PI-RADS 4 lesions demonstrated the highest upstaging rate (43.5%), exceeding both PI-RADS 3 (33.3%) and PI-RADS 5 (18.2%) lesions. This counterintuitive pattern has several potential explanations. PI-RADS 5 lesions may receive more aggressive and targeted biopsy approaches, leading to better initial grade characterization (i.e., the worst PCa growth pattern is identified on biopsy). In contrast, PI-RADS 4 lesions may represent a distinct biological subset with greater intratumoral heterogeneity, increasing the chance of missing higher-grade foci at biopsy and making accurate grade assessment more challenging despite adequate sampling. This finding has important clinical implications, suggesting that PI-RADS 4 lesions should not be considered “intermediate risk” for upstaging purposes and may warrant more aggressive biopsy strategies or closer surveillance protocols [25].

The 69.4% upstaging rate among Grade 1 patients represents one of the highest reported in contemporary literature, with particularly concerning rates in the MRI subcohort (85.7%), with the caveat of its small sample size (7 cases) and the overall selection bias for this particular Grade 1 subpopulation. Herein, these seven patients either underwent MRI due to clinical suspicion of more aggressive disease (e.g., rising PSA, suspicious DRE) and/or underwent RRP, albeit for apparently low-grade disease, due to signs of increased oncological risk on imaging. Therefore, this seemingly paradoxical increase in ISUP upstaging frequency among MRI-evaluated Grade 1 patients does not necessarily contradict MRI’s clinical value and should be interpreted cautiously, given the small sample and selection factors involved.

Even so, this finding may potentially have profound implications for treatment decision-making protocols and challenges current risk stratification paradigms overall, reflecting the well-documented issue of accurately identifying low-grade disease on limited biopsy sampling [26]. In fact, the European Association of Urology guidelines currently recommend mpMRI before biopsy and/or enrollment in active surveillance protocols [27], a recommendation strongly supported by our data. Conversely, the elevated upstaging rates may also reflect several factors specific to our regional healthcare environment: potential differences in biopsy technique or adequacy compared to high-volume international centers, possible variations in pathological interpretation, and patient selection factors, wherein only higher-risk Grade 1 patients proceeded to MRI and surgery.

PSA density emerged as the most influential predictor across all analyses (SHAP importance: 0.287), consistent with extensive literature supporting its role in PCa risk assessment [28,29]. Our identified threshold of 0.20 ng/mL^2^ aligns well with international recommendations. The SHAP analysis revealed important non-linear relationships, showing an additional threshold effect at approximately 0.25 ng/mL^2^, above which upstaging risk increased exponentially. This finding suggests a clinically actionable cutoff that could support intensified treatment decisions and highlights the importance of interpreting PSA density as a continuous, not binary, variable. The pathophysiological basis for PSA density’s predictive power likely reflects the relationship between tumor volume, grade, and PSA production—with higher-grade tumors producing more PSA per unit volume of prostate tissue, making density a more accurate reflection of tumor biology than absolute PSA values alone. Conversely, very aggressive, dedifferentiated tumors may confoundingly lose the ability to generate PSA, with locally advanced PCa patients presenting, albeit rarely, with low to even normal total PSA serum values.

Our machine learning analysis demonstrated superior performance of GBMs (AUC = 0.812) compared to traditional logistic regression (AUC = 0.721), representing a clinically meaningful 13% improvement in discrimination. This translates to correctly identifying eight additional upstaging cases per 100 patients at typical clinical decision thresholds. More importantly, the integration of SHAP analysis represents a paradigm shift toward explainable AI in clinical medicine. SHAP enables patient-specific risk assessment by quantifying each factor’s contribution to upstaging probability, revealing synergistic effects between variables that would be missed by traditional approaches, and helps clinicians understand exactly why a model recommends certain actions, building trust and facilitating implementation. However, while our models were internally validated, external validation on larger, multicenter cohorts will be necessary to confirm generalizability.

Our upstaging rate of 38.7% falls within the expected range but toward the higher end of contemporary series. Recent studies report rates of 30–40% [3,4,9], suggesting lingering potential opportunities for improvement in PCa diagnostic accuracy. Resource constraints affecting MRI availability (36.6% utilization) may impact overall diagnostic accuracy, as international centers with routine pre-biopsy MRI report lower upstaging rates [30,31,32]. Variations in Gleason grading between institutions remain problematic despite standardization efforts [33], and implementation of AI-assisted grading systems might improve consistency and reduce upstaging rates [34].

ISUP downstaging occurred in about 10% of patients, a rate consistent with the existing literature. This indicates that a subset of men may have had their disease grade initially overestimated on biopsy, thus potentially leading to unnecessary definitive treatment, i.e., overtreatment. Notably, we observed that older patients were more prone to downgrading, possibly due to age-related variations in tumor biology and/or biopsy sampling issues, although our sample is too limited to draw firm conclusions. The similar downgrading rates between the MRI and non-MRI cohorts (≈10% each) suggest inherent challenges in accurate grade assessment despite modern techniques, i.e., even with modern imaging, some grade overestimation is unavoidable. Recognizing the possibility of ISUP overestimation on initial biopsy is important in counseling patients—it underscores the need to balance the risk of missing aggressive disease (upstaging) against the risk of overtreating indolent disease (downgrading).

The clinical and economic implications of accurate upstaging prediction extend beyond individual patient care. Our analysis revealed strong associations between upstaging and adverse pathological features: ECE occurred in 52.7% of upstaged patients versus 28.7% with concordant grading (*p* = 0.008), while positive surgical margins were found in 38.2% versus 21.8% (*p* = 0.045), lymphovascular invasion in 23.6% versus 12.3% (*p* = 0.126), and perineural invasion in 58.2% versus 39.7% (*p* = 0.076). These findings suggest that upstaging identifies biologically aggressive tumors prone to local advancement, supporting intensified treatment approaches such as extended lymphadenectomy, wider excision margins, or consideration of multimodal therapy in high-risk patients. Conversely, preventing unnecessary active surveillance in six to seven patients per 100 (extrapolating based on our MRI data) generates substantial healthcare savings while improving oncological outcomes when accounting for delayed treatment, additional biopsies, and progression management [35]. The superior performance of PSA density-based models suggests that meaningful improvements in upstaging prediction can be achieved with readily available clinical parameters, making these approaches feasible even in resource-constrained settings like Romania.

Several limitations warrant acknowledgment. The single-center design may limit generalizability, particularly given institutional variations in technique and patient populations. The retrospective nature introduces potential selection bias, as surgical patients may differ systematically from those choosing alternative treatments. Notably, because our cohort included only surgically treated patients, our findings do not directly measure outcomes of active surveillance. Thus, caution is warranted when extrapolating our results to active surveillance populations. Furthermore, the MRI subcohort of 52 patients, while adequate for analysis, had limited statistical power to detect significant differences. Future multicenter collaborations should prioritize larger cohorts, enabling robust validation of MRI benefits [36]. The machine learning models require external validation before clinical implementation, and future studies should investigate incorporating genomic classifiers, novel imaging biomarkers, and liquid biopsy platforms to further enhance prediction accuracy [37,38].

Despite these limitations, our study possesses several notable strengths. The comprehensive data collection encompassed clinical, imaging, and detailed histopathological parameters, providing a more complete picture of factors influencing grade migration. The 4-year recruitment period ensures contemporary practice patterns, including modern biopsy techniques and current grading standards. Our rigorous statistical methodology with bootstrap validation, decision curve analysis, and SHAP interpretability provides robust performance estimates and clinical contextualization often lacking in prediction model studies. Furthermore, the inclusion of both upstaging and downstaging outcomes offers a more nuanced understanding of grade discordance than traditional unidirectional analyses.

This study represents one of the first comprehensive analyses of ISUP upstaging in a Romanian PCa cohort, providing valuable insights for national healthcare planning. Moreover, this study is the first to apply SHAP explainability to ISUP grade group discordance between biopsy and RP in PCa, revealing clinically relevant risk synergies and underscoring the potential of integrating advanced analytics into prognostic tools. The relatively high upstaging rates reported suggest opportunities for improvement through development of national protocols for biopsy technique, pathological interpretation, and MRI utilization; enhanced training for urologists, radiologists, and pathologists in modern PCa diagnostics; strategic investment in MRI infrastructure and AI-assisted diagnostic tools; and implementation of quality metrics and benchmarking programs to drive continuous improvement in diagnostic accuracy and clinical outcomes.

Future efforts should focus on external validation of our model in larger, multicenter cohorts and on the seamless integration of such tools into clinical practice. In particular, the development of user-friendly clinical decision support systems (e.g., risk calculators or electronic health record-integrated alerts) incorporating our machine learning model’s predictions—alongside the explanatory insights from SHAP—would facilitate widespread implementation. By providing an individualized risk estimate and the rationale behind it, these tools can assist clinicians in making informed decisions (for example, identifying biopsy Grade 1 patients who should be re-evaluated or treated due to high upstaging risk). Ultimately, integrating explainable AI models into the pre-surgical workflow could improve PCa patient counseling and personalize management strategies.

## 5. Conclusions

ISUP grade group migration affects nearly half (48.6%) of PCa patients in our Romanian cohort, with 38.7% experiencing upstaging and 9.9% experiencing downstaging. This bidirectional grade migration provides a more complete picture of biopsy-to-RP grade discordance than previously reported. The clinically promising 6.5% absolute reduction in upstaging among patients undergoing preoperative MRI, though not statistically significant, supports continued investment in MRI infrastructure.

The exceptionally high upstaging rate among Grade 1 patients (69.4%) raises serious concerns about the safety and accuracy of current therapeutic decision-making and thus emphasizes the need for enhanced risk stratification in this apparently (very) low-risk PCa group. Conversely, the 30% combined up/downstaging rate in Grades 3–4 highlights substantial grading uncertainty in intermediate and high-risk disease, with implications for treatment intensity decisions.

PSA density emerges as the most influential predictor across all patients (SHAP importance: 0.287), supporting its routine incorporation into clinical decision-making. The unexpected finding that PI-RADS 4 lesions demonstrate higher upstaging rates than PI-RADS 5 lesions (43.5% vs. 18.2%) challenges conventional risk stratification and warrants further investigation.

Machine learning approaches, particularly gradient boosting with SHAP analysis, provide superior predictive performance (AUC = 0.812 vs. 0.721 for traditional logistic regression) while offering unprecedented interpretability for clinical implementation. The integration of SHAP analysis represents a paradigm shift toward explainable AI in clinical medicine, enabling transparent model interpretation that builds clinician trust and facilitates adoption.

These findings have important implications for Romanian healthcare policy and resource allocation. The development of standardized protocols for biopsy technique and pathological interpretation, enhanced training programs, and strategic technology investments could significantly improve diagnostic accuracy and potentially prevent undertreatment of aggressive PCas, while also avoiding overtreatment of indolent ones. The identification of bidirectional grade migration emphasizes the need for quality assurance programs and consideration of centralized pathology review for optimal patient care.

Future efforts should focus on external validation in larger multicenter cohorts, integration of emerging biomarkers, and prospective evaluation of model-guided treatment strategies. The development of user-friendly clinical decision support tools incorporating SHAP explanations would facilitate widespread implementation and improve PCa care across diverse healthcare settings.

## Figures and Tables

**Table 1:** Baseline characteristics of the study cohort stratified by MRI status and ISUP grade migration outcome.

Characteristic	Total (*n* = 142)	Non-MRI (*n* = 90)	MRI (*n* = 52)	*p*-Value	Upstaging (*n* = 55)	No Change (*n* = 73)	Downstaging (*n* = 14)	*p*-Value
**Clinical Parameters**								
Age, years	64.5 ± 5.5	64.6 ± 5.4	64.5 ± 5.7	0.931	65.1 ± 5.8	64.2 ± 5.3	68.1 ± 4.9	0.124
PSA, ng/mL	9.0 (6.8–13.8)	9.0 (6.8–13.8)	9.1 (6.8–13.8)	0.876	10.8 (7.5–15.2)	8.5 (6.2–12.8)	8.7 (6.0–13.5)	0.041
Prostate volume, mL	41.8 ± 14.6	42.1 ± 14.8	41.3 ± 14.2	0.751	39.4 ± 13.4	43.2 ± 15.1	44.1 ± 16.2	0.287
PSA density, ng/mL^2^	0.233 (0.162–0.326)	0.230 (0.160–0.328)	0.237 (0.164–0.324)	0.814	0.257 (0.179–0.398)	0.229 (0.158–0.301)	0.216 (0.145–0.298)	0.024
Abnormal DRE	119 (83.8%)	71 (78.9%)	48 (92.3%)	0.041	45 (81.8%)	62 (84.9%)	12 (85.7%)	0.872
**MRI Parameters (*n* = 52)**								
PI-RADS score								0.412
3	-	-	15 (28.8%)		5 (27.8%)	7 (24.1%)	3 (60.0%)	
4	-	-	23 (44.2%)		10 (55.6%)	11 (37.9%)	2 (40.0%)	
5	-	-	11 (21.2%)		2 (11.1%)	9 (31.0%)	0 (0%)	
Number of lesions	-	-	1.4 ± 0.6		1.5 ± 0.7	1.3 ± 0.5	1.2 ± 0.4	0.541
**Biopsy Parameters**								
Total cores	12.5 ± 1.9	12.4 ± 1.8	12.7 ± 2.1	0.352	12.8 ± 2.2	12.3 ± 1.7	12.1 ± 1.8	0.287
Positive cores	4.9 ± 3.3	4.8 ± 3.2	5.1 ± 3.5	0.621	6.2 ± 3.7	4.2 ± 2.8	3.8 ± 2.9	<0.001
Positive core ratio	0.39 ± 0.25	0.39 ± 0.24	0.40 ± 0.26	0.798	0.48 ± 0.27	0.34 ± 0.22	0.31 ± 0.23	0.003
Initial ISUP Grade								<0.001
Grade 1	36 (25.4%)	29 (32.2%)	7 (13.5%)		25 (45.5%)	11 (15.1%)	0 (0%)	
Grade 2	63 (44.4%)	35 (38.9%)	28 (53.8%)		19 (34.5%)	43 (58.9%)	1 (7.1%)	
Grade 3	25 (17.6%)	14 (15.6%)	11 (21.2%)		6 (10.9%)	13 (17.8%)	6 (42.9%)	
Grade 4	13 (9.2%)	8 (8.9%)	5 (9.6%)		5 (9.1%)	2 (2.7%)	6 (42.9%)	
Grade 5	5 (3.5%)	4 (4.4%)	1 (1.9%)		0 (0%)	4 (5.5%)	1 (7.1%)	

Data presented as mean ± SD, median (IQR), or *n* (%). DRE = digital rectal examination; PSA = prostate-specific antigen; PI-RADS = Prostate Imaging Reporting and Data System; ISUP = International Society of Urological Pathology.

**Table 2:** Univariate predictors of ISUP grade group migration outcomes.

Variable	Upstaging vs. No Change		Downstaging vs. No Change		Any Migration vs. No Change	
	OR (95% CI)	*p*-Value	OR (95% CI)	*p*-Value	OR (95% CI)	*p*-Value
Age (per 5 years)	1.18 (0.85–1.64)	0.321	1.89 (1.11–3.21)	0.019	1.29 (0.95–1.75)	0.102
PSA (per 5 ng/mL)	1.24 (1.02–1.51)	0.029	1.05 (0.75–1.47)	0.774	1.20 (1.00–1.44)	0.048
Prostate volume (per 10 mL)	0.83 (0.65–1.06)	0.139	1.04 (0.69–1.57)	0.848	0.87 (0.69–1.09)	0.226
PSA density > 0.20 ng/mL^2^	1.73 (0.89–3.36)	0.106	0.67 (0.21–2.14)	0.498	1.44 (0.77–2.69)	0.252
Abnormal DRE	0.81 (0.37–1.77)	0.604	1.06 (0.23–4.90)	0.940	0.85 (0.40–1.79)	0.669
Positive cores (per core)	1.19 (1.07–1.33)	0.002	0.93 (0.77–1.12)	0.437	1.13 (1.02–1.25)	0.018
Positive core ratio ≥ 33%	0.97 (0.50–1.88)	0.931	0.62 (0.20–1.92)	0.406	0.89 (0.48–1.65)	0.711
UCSF-CAPRA score (per point)	1.25 (1.06–1.47)	0.008	1.18 (0.88–1.58)	0.269	1.23 (1.06–1.43)	0.006
D’Amico intermediate/high	1.89 (0.97–3.68)	0.061	3.64 (0.45–29.34)	0.225	2.08 (1.09–3.96)	0.026
**MRI parameters (*n* = 52)**						
PI-RADS score						
3 (reference)	1.00	-	1.00	-	1.00	-
4	1.64 (0.41–6.56)	0.482	1.64 (0.13–21.0)	0.698	1.64 (0.44–6.11)	0.462
5	0.51 (0.08–3.49)	0.496	2.00 (0.14–28.8)	0.611	0.86 (0.17–4.27)	0.851
Lesion diameter > 1.5 cm	1.01 (0.32–3.22)	0.984	2.85 (0.44–18.4)	0.268	1.27 (0.45–3.60)	0.654
Multiple lesions	0.43 (0.08–2.26)	0.318	1.01 (0.09–11.2)	0.994	0.52 (0.12–2.21)	0.374
ECE on MRI †	0.81 (0.07–9.71)	0.870	2.11 (0.11–41.2)	0.623	1.14 (0.15–8.72)	0.903
SVI on MRI †	1.59 (0.09–27.0)	0.750	10.3 (0.44–242)	0.149	3.78 (0.31–46.1)	0.294

† Interpret with caution due to low event rates (extracapsular extension—ECE: *n* = 4, seminal vesicle invasion—SVI: *n* = 3). OR = odds ratio; CI = confidence interval; MRI = magnetic resonance imaging; PSA = prostate-specific antigen; PI-RADS = Prostate Imaging Reporting and Data System; DRE = digital rectal examination; UCSF-CAPRA = The University of California, San Francisco-Cancer of the Prostate Risk Assessment.

**Table 3:** Multivariate predictors of ISUP grade group upstaging.

Variable	Adjusted OR (95% CI)	*p*-Value
PSA density > 0.20 ng/mL^2^	1.89 (0.91–3.93)	0.090
Positive cores (per core)	1.17 (1.04–1.31)	0.009
UCSF-CAPRA score (per point)	1.19 (1.00–1.42)	0.049

Model performance: AUC = 0.721 (95% CI: 0.631–0.811), Hosmer–Lemeshow *p* = 0.341.

**Table 4:** Comparative performance of prediction models for ISUP grade group upstaging.

Model	AUC (95% CI)	Sensitivity	Specificity	PPV	NPV	Accuracy
Logistic Regression	0.721 (0.631–0.811)	65.5%	71.3%	57.1%	78.0%	68.8%
LASSO-penalized Logistic Regression	0.758 (0.671–0.845)	69.1%	74.7%	61.3%	80.6%	72.3%
Random Forest	0.794 (0.712–0.876)	72.7%	78.2%	66.7%	82.9%	75.8%
Gradient Boosting	0.812 (0.735–0.889)	76.4%	80.5%	70.0%	85.4%	78.9%

AUC = area under the receiver operating characteristic curve; CI = confidence interval; PPV = positive predictive value; NPV = negative predictive value.

**Table 5:** Pathological outcomes stratified by ISUP grade group migration status.

Outcome	Upstaging (*n* = 55)	No Change (*n* = 73)	Downstaging (*n* = 14)	*p*-Value
pT Stage				0.024
- pT2	26 (47.3%)	52 (71.2%)	10 (71.4%)	
- pT3a	23 (41.8%)	17 (23.3%)	3 (21.4%)	
- pT3b	6 (10.9%)	4 (5.5%)	1 (7.1%)	
Positive surgical margins	21 (38.2%)	16 (21.9%)	3 (21.4%)	0.089
Lymphovascular invasion	13 (23.6%)	9 (12.3%)	1 (7.1%)	0.126
Perineural invasion	32 (58.2%)	29 (39.7%)	5 (35.7%)	0.076
Lymph node involvement *	3/42 (7.1%)	2/51 (3.9%)	0/11 (0%)	0.514

* Denominator represents patients who underwent lymphadenectomy. Data presented as *n* (%).

## Data Availability

The data presented in this study are available on request from the corresponding author.

## Abbreviations

AI	Artificial Intelligence
AUC	Area Under the Curve
CI	Confidence Interval
DRE	Digital Rectal Examination
ECE	Extracapsular Extension
GBM	Gradient Boosting Model
IQR	Interquartile Range
ISUP	International Society of Urological Pathology
LR-LASSO	Logistic regression with LASSO regularization
mpMRI	Multiparametric Magnetic Resonance Imaging
MRI	Magnetic Resonance Imaging
NPV	Negative Predictive Value
OR	Odds Ratio
PCa	Prostate Cancer
PI-RADS	Prostate Imaging-Reporting and Data System
PPV	Positive Predictive Value
PSA	Prostate-Specific Antigen
RFM	Random Forest Model
ROC	Receiver Operating Characteristic
RP	Radical Prostatectomy
RRP	Open Radical Retropubic Prostatectomy
SD	Standard Deviation
SHAP	SHapley Additive exPlanations
SVI	Seminal Vesicle Invasion
UCSF—CAPRA	University of California, San Francisco—Cancer of the Prostate Risk Assessment
VIF	Variance Inflation Factor
